# Comparison of microleakage between different restorative materials to restore marginal gap at crown margin

**DOI:** 10.7717/peerj.10823

**Published:** 2021-02-25

**Authors:** Satheesh B. Haralur, Ghaseb Ahmed AL Ghaseb, Norah Ali Alqahtani, Bader Alqahtani

**Affiliations:** Department of Prosthodontics, College of Dentistry, King Khalid University, Abha, Asir, Saudi Arabia

**Keywords:** Secondary caries, Full veneer crowns, Interfacial micro-leakage, Dental Restorations, Restoration margin caries

## Abstract

**Background:**

An occurrence of secondary caries around the indirect restoration margin is reported to remain a leading cause of failures.

**Objective:**

This study aimed to test the interfacial microleakage of conventional glass-ionomer (CGI), resin-modified glass ionomer (RMGI) and Nano-hybrid composite (CR) restorations at a full veneer margin crown.

**Methods:**

Ninety human extracted molar teeth were divided into three groups (*n* = 30). Each group was subdivided into three subgroups (*n* = 10) according to the extent of the structural defects; The structural defect in G1 had a depth of 1.5 mm, width and length at 2 mm and 1 mm intrusion within the crown cervical margin. The corresponding structural defect dimension values for G2 were 2, 5, 4 and 2 mm with defects extending onto the root structure. Meanwhile, G3: structural deficiency of 2 mm depth, 3 mm width and 3 mm length and with 1.5 mm extension into the prepared teeth. These structural defects in each subgroup were restored with CGI, RMGI and CR. Artificial carious lesion formation was induced at the cervical finish line with a demineralizing solution. The artificial carious lesions were restored as per the group distribution. Subsequently, teeth samples were prepared and cemented with Nickel-chromium full coverage restorations utilizing glass-ionomer luting cement. Teeth samples were thermocycled, isolated with nail varnish, and immersed in 0.1% methylene blue for 24 h. The teeth samples were sectioned longitudinally, dye penetration was evaluated with a stereomicroscope. The data were analyzed with Kruskal–Wallis and Mann–Whitney *U* tests.

**Results:**

CGI-G1 recorded the highest micro-leakage score at 1.450; while CR-G3 recorded the least score (0.350). At a cementum-restoration interface, CR-G1 (0.850) documented the lowest micro-leakage; RMGI-G3 had a greater value at 1.700.

**Conclusions:**

The hybrid CR could be effectively used to restore the restoration of a marginal gap around crown margins.

## Introduction

The full coverage restorations (FCR) fabricated from different materials are utilized to protect teeth with extensive restoration or root canal treated, and as the retainers for tooth-supported fixed partial dentures (FPD). Clinicians also use them for various occlusal and aesthetic rehabilitation purposes. Previous epidemiological studies show a considerable percentage of populations had teeth restored with FCR. The research in the UK (Nuttall et al., 2001) reported one-third of dentate adults had at least single crowned teeth, while Lundegren et al. (2019) reported 40% of individuals with a fixed prosthesis rehabilitation. Research (Peeran et al., 2016) on the Saudi Arabia population showed that approximately 56.4–57.2% of subjects needed fixed Prosthodontic treatment. Besides increased awareness, the larger economically empowered sections of the community would like to maintain a functional and aesthetically acceptable dentition for a more extended duration (Manski & Meyerhoefer, 2017). Along with the increasingly aging dentate population across the nations, the researchers predict the greater demand for dental services (Maia et al., 2018) and indirect restorations.

Maintaining a functional fixed prosthesis in middle-aged and older adults is a new area of challenge for dentists. The principal reason for failures in crowns and FPD are biological complications like caries, periodontal diseases, and loss of pulp vitality (Limones et al., 2020). The biological failures among FPD are estimated at 85.5–92.8%, depending upon the number of abutments (Al Refai & Saker, 2018). Goodacre et al. (2003) observed common clinical complications in fixed prosthodontics were caries in abutments at 18%, while 11% of these abutments required endodontic treatment. Pjetursson et al. (2015) described a significantly higher incidence of caries in abutment as a primary reason for failure. Investigators attribute the increased susceptibility of crowns margin for secondary caries to rough, overhanging, inadequately extended, or poorly adapted margins. The tooth-restoration interface hastens the accumulation of biofilm mass and hinders its removal. The micro-environmental conditions like predominant anaerobic bacteria, lack of salivary buffering, and low pH equally contribute to increasing caries susceptibility at subgingival crown margins (Dhanraj, Anand & Ariga, 2013). Secondary caries around crown margins leads to multiple complications, including pain, sensitivity, unpleasant taste, foul odor, pulpal/periodontal diseases, abutment fracture, and discoloration of restoration.

The researchers suggest multiple treatment options, including teeth extraction, repair by direct restorations with/without removing the crown and remake the crown. There is inadequate evidence in dental literature regarding the potential for repairing than replacing the crown with secondary caries at its margins. The location and extent of cervical defects determine the cavity surface with only enamel or combined enamel-dentin substrates. The structural difference between enamel and dentin surfaces influences the chemical and mechanical bond advancement with restorations (Ageel & Alqahtani, 2019). Hence, the secondary caries location and its extent define the management methods (Alomari et al., 2009). Therefore, it is prudent to ascertain the feasibility of different restorative materials to repair the cervical carious lesions in various dimensions. The secondary caries restoration with direct restorative materials possesses multiple advantages like short clinical time, cost-effectiveness, and feasible provisional treatment for a moderate duration. The marginal adaptation of restoration to the tooth structure is a critical factor in determining the longevity of clinical service (Hepdeniz & Ermis, 2019). Inadequate marginal seal enhances plaque accumulation, dissolution of luting cement, and micro-leakage (Larson, 2012). The micro-leakage is a primary cause for FCR failures, leading to hypersensitivity, margin discoloration, and secondary caries (Santos & Busato, 2019). There is a need for studies to further the evidence to assess the effectiveness of marginal repair as a suitable treatment option for the management of the crown with marginal caries. The knowledge would assist the dentist to make an informed decision to develop an appropriate treatment plan, communicate the treatment protocol, and realistic treatment outcome to the patient. The study tested commonly used direct restorative materials like conventional glass ionomer (CGI), Resin-modified glass ionomer (RMGI) and composite (CR) to repair the carious lesion at the crown margin. This in-vitro study assessed the hypothesis that direct restorative materials as effective treatment options for the management of secondary caries around the crown margin. The study aim included the evaluation of the micro-leakage in different restorative materials used to restore the teeth defect of various configurations around the crown margin.

## Materials and Methods

The institutional ethical review board at the College of Dentistry, King Khalid University approved this study protocol (SRC/ETH/2017-18/083). All procedures and experiments in this research were performed following ethical guidelines and regulations of the College of Dentistry, King Khalid University. Teeth samples utilized in the study were extracted for periodontal or orthodontic reasons. The patients were explained about using their teeth for research purposes, and written informed consent was obtained. Ninety freshly extracted molar teeth were collected and stored in distilled water for a maximum of 2 months until the preparation for the study (Komabayashi et al., 2009). The teeth used in the study were evaluated under X 5 magnification to eliminate the samples with micro-cracks. The exclusion criteria for the teeth samples included root or coronal caries, fractures, previous endodontic treatment, and internal resorption. The caries detection in the samples was accomplished with a visuo-tactile method by using explorer and Periapical radiographs.

**Figure 1:**
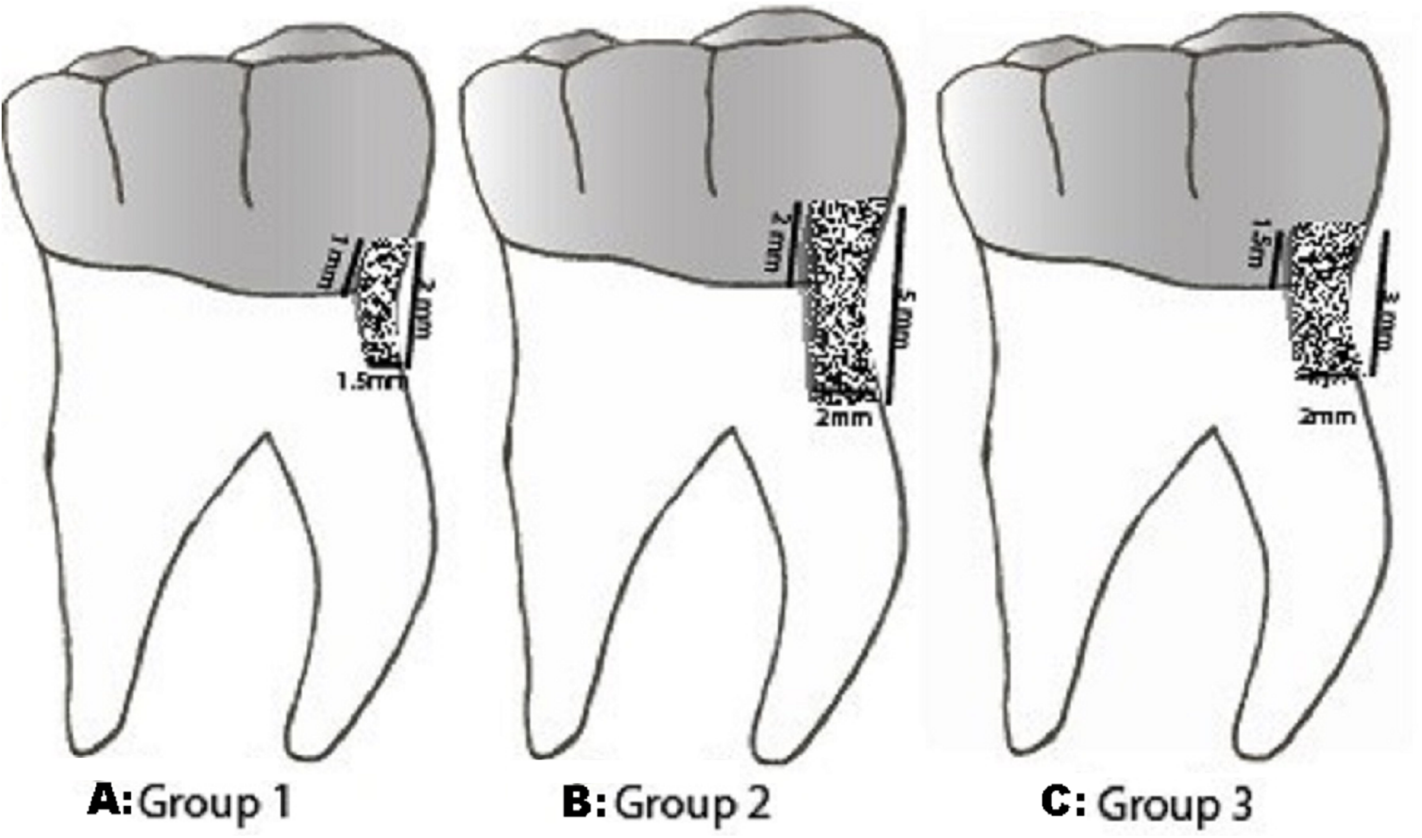
Group of secondary caries. Description of structural defects in different groups. (A) Group 1 - 1.5 mm D × 2 mm W × 2 mm L and 1 mm within proposed FCR. (B) Group 2 - 2 mm D × 5 mm W × 4 mm L and 2 mm within crown margin (C) Group 3 - 2 mm D × 3 mm W × 3 mm L with 1.5 mm extension within the crown margin.

The Teeth samples were randomly divided into three groups: each with 30 samples. Each group was designated according to the dimension and extension of tooth structure defect accomplished with the demineralizing solution. The proposed cervical finish line was marked on the tooth approximately 1 mm coronal to the cementoenamel junction. It was marked in indelible ink during the demineralizing process. G1: (Fig. 1A) structural defect with 1.5 mm depth, 2 mm width and 2 mm length, beside one mm extension above the proposed cervical finish line. G2: (Fig. 1B) tooth structure defect measuring 2 mm depth, 5 mm length and 4 mm width, 2 mm intrusion inside the crown margin. This group had the extension of defects over the root surface. G3: (Fig. 1C) structural defect extensions of 2 mm depth, 3 mm width and 3 mm length and with 1.5 mm extension into the prepared teeth. Each group was further divided into (*n* = 10) three sub-groups to be restored with CGI, RMGI and restorative Nanohybrid Composite (CR). Based on previously published studies (Ayna et al., 2018; Garcia Mari, Climent Gil & Puy, 2019), effect size (d) 1.3, α at 0.05, and 1 − β(power) at 0.85 the sample size was determined to be 10 per sub-group, resulting in a total of 30 specimens per group. The sample size was calculated with the G*Power software (version 3.1; University of Dusseldorf) (Faul et al., 2009).

Artificial carious lesion formation was induced with a demineralizing solution (Buckshey et al., 2019; Freitas et al., 2018) in the proximal regions. The demineralizing solution contained CaCl_2_2.2mM, NaH_2_PO_4_ 2.2mM, and 0.05M acetic acid, which was adjusted with KOH 1M to maintain the pH at 5.0. The localized area for demineralization was exposed to demineralization, and the intact surface was covered with two layers of nail varnish (Revlon, New York, NY, USA). The teeth samples were immersed within the demineralizing solution for seven days. The pH was monitored daily and sustained by an addition of 10 M KOH. After seven days of demineralization, the teeth samples were washed with de-ionized water and proceeded for preparation.

The root surfaces of all the samples 5 mm below the cementoenamel junction were covered by two layers of adhesive tape and embedded in PVC tubes using non-shrink epoxy resin. Five mm distance between cement/enamel junction and embedding resin was maintained during embedding of samples. The adhesive tapes were to enable the easy removal of teeth samples to post crown preparation. All the teeth samples were prepared with medium grit diamond bur for the full veneer metal crowns with 0.6 mm deep chamfer finish line and 6 degrees taper. Crown preparation for all the teeth samples was accomplished under uninterrupted water coolant spray. The preparation and taper were standardized with the help of a parallel milling machine. The coronal height was kept at 5 mm. The defects around the finish line margin simulating secondary caries with a different configuration were refined with round tungsten carbide. The defects around the cervical margins were prepared before crown cementation to serve in the uniformity of depth, location, and width of the lesion within the crown margin. All the dimensions were measured and standardized with the digital caliper (fino Pra Ceci caliper, FINO GmbH, Mangelsfeld18 Bad Bocklet Germany). The graduated periodontal probe was used to confirm the depth of the cavity. All the tooth preparation and restoration were performed by a single investigator.

**Figure 2:**
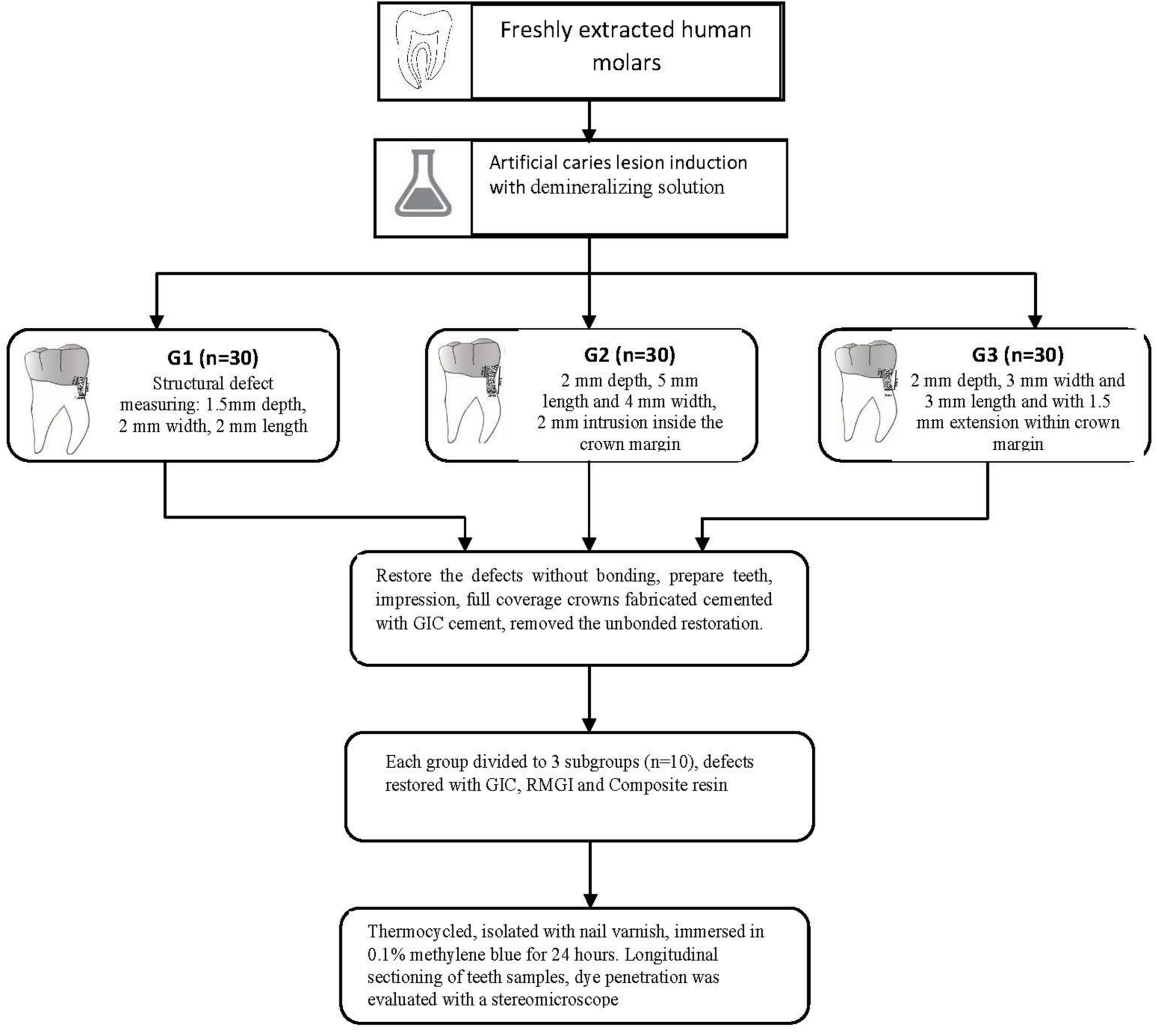
Criteria for evaluation of microleakage.

The structural defects were restored with composite resin without a bonding agent application. This was to facilitate its easy removal after crown cementation. Finally, the preparation and finish line were refined. The primary improved stone die was obtained from an irreversible hydrocolloid impression in a metal stock tray. The auto polymerizing acrylic special tray was fabricated after adapting uniform 2 mm wax relief on the occlusal and axial surfaces. The final impression was made in additional polyvinyl silicone impression materials (Reprosil, Dentsply Sirona, Charlotte, NC, USA). The working die was fabricated in type IV stone (New Fujirock, GC Corporation Bunkyo-Ku, Tokyo, Japan). Subsequently, Nickel-Chromium crowns were fabricated in a conventional method and cemented with Glass Ionomer-Type I (Riva, SDI, Inc. Bristol, PA, USA) luting, self-curing cement. Before the cementation of the crown, petrolatum jelly was applied to the area corresponding to the composite restoration at prepared structural defects. The cement capsules were activated and triturated for 10 s in an amalgamator. Intaglio surface of the crown was coated with cement and filled not more than half. The crown was seated on the tooth sample, held under steady pressure, and the excess cement was removed after 2 min at the gel phase using a hand scaler. Following the complete set of luting cement; unbonded composite restoration from marginal structural defects was extricated with the help of small spoon excavators. This was to simulate the clinical practice of restoring the structural defect around the crown margin while crown in situ (Fig. 2). The Intaglio surface of the crowns corresponding to the defect site was cleaned and air-abraded with 50 M alumina oxide using intra-oral sandblaster (MicroEtcher II, Danville Materials, Patterson Companies, Saint Paul, MN, USA). Prepared teeth samples from each group were randomly divided into three subgroups (*n* = 10). The CGI, RMGI and restorative Nanohybrid Composite (CR) were used to restore the samples from each subgroup. The restorative procedure is described in Table 1.

**Table 1:** Description of material and procedure of restorations used in the study.

Group	Material	Manufacturer	Procedure
A	Conventional Glass-ionomer (CGI)	Ketac fil Plus aplicap, 3M Deutschland Gmbh, Carl-Schurz, Neuss, Germany	No Pre-conditioning, mix the capsule after activation for 10 s, application of glaze and cure it for 10 s.
B	Resin modified glass ionomer (RMGI)	Riva light cure, SDI limited, Bayswater, Victoria, Australia	Surface etching with 37% phosphoric acid for 5 s, Rinsed with water and blot dried, a single coat of a Scotch bond Universal Adhesive is applied and dried. The RGMI capsule activated and light cured (Elipar S10, 3M, ESPE, St. Paul, MN, USA) for 20 s.
C	Nonohybrid composite restoration (CR)	Tetric N-Ceram, Ivoclar Vivodent AG, Schaan, Liechtenstein	Surface etching with 37% phosphoric acid for 15 s,wash, blot dried. Single coat application of Scotch bond Universal Adhesive, air dried for 5 s. Restored with composite and light cured for 20 s.

**Figure 3:**
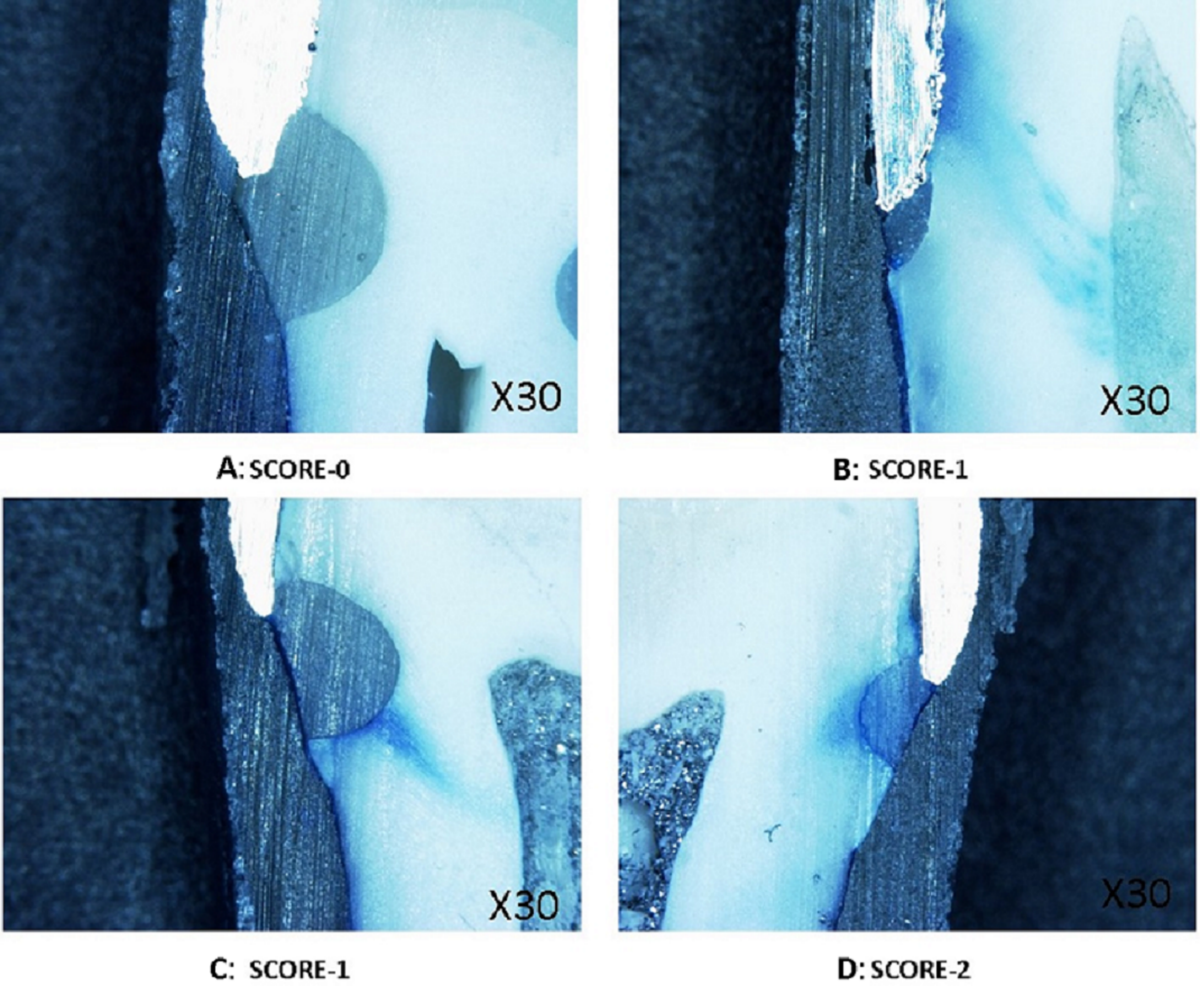
Steriomicrospe images for microleakage. (A) Score 0-no dye penetration. (B) Score 1-dye diffusion along the gingival wall. (C) Score -1. (D) Score -2 Dye infiltration in both gingival and axial wall.

**Figure 4:**
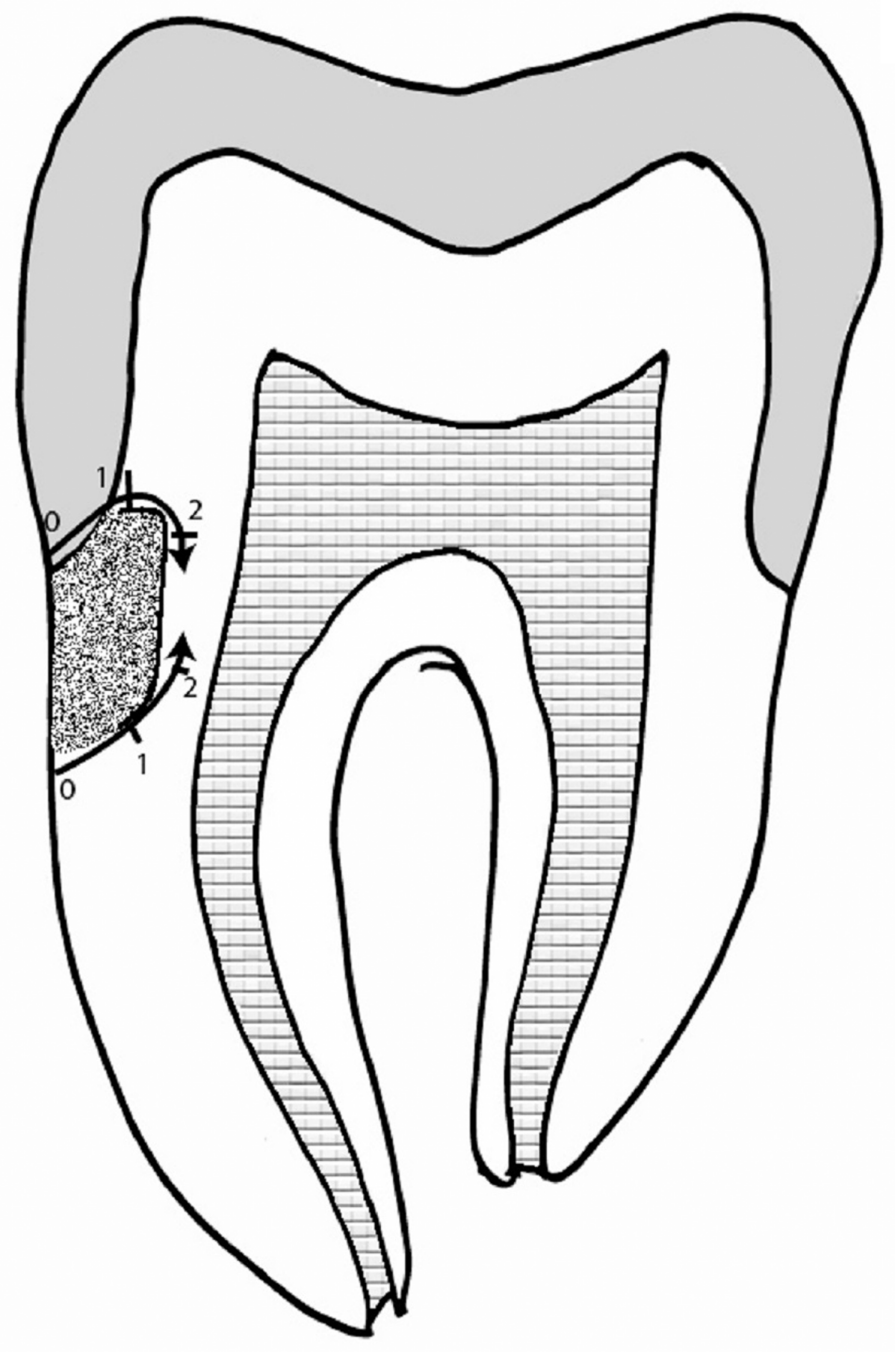
Microleakage score.

Teeth samples were subjected to accelerated aging by thermocycling between 5 °C and 55 °C for 1,500 cycles with a dwelling time of one minute (Thermocycler, SD Mechatronik, Feldkirchen-Westerham Germany). The apical foramen was sealed with composite resin, and all surfaces were coated with two layers of nail varnish except the 1 mm around the restorations. The micro-leakage was accessed with a dye penetration test by 0.1% methylene blue immersion for 24 h. Subsequently, teeth samples were washed in running water, lightly pumiced. The teeth samples were sectioned in the mesiodistal direction at the center of the restoration with the help of a 0.5 mm low-speed diamond disc. The Sectioned teeth samples were appraised with a stereomicroscope at 30×*g* magnification to measure the depth of dye penetration (Figs. 3A–3D). The following criteria (Fig. 4) were observed for scoring micro-leakage at both Cementum-restoration (C-R) and Crown-restoration(C-R) interfaces: no penetration of dye (0); penetration of dye along the gingival wall (1); penetration including gingival margin and an axial wall (2). Acquired data were evaluated with SPSS 19 (IBM Corporation, Armonk, New York, NY, USA). The micro-leakage between groups was assessed with Kruskal–Wallis and Mann–Whitney *U* test, and the level of significance was at *p* = 0.05.

## Results

All the restorations tested in the study showed a varying range of micro-leakage at both interfaces. The micro-leakage at Crown-restoration and Cementum-restoration interfaces for all restorations are presented in Table 2.

**Table 2:** Mean interfacial micro leakage scores for all groups.

Groups	Crown-restoration (Mean ± SD)	Cementum-restoration (Mean ± SD)
RMGI-G1	0.90 (0.71)	1.100 (0.820)
RMGI-G2	0.850 (0.670)	1.40 (0.753)
RMGI-G3	0.850 (0.812)	1.700 (0.571)
CGI-G1	1.450 (0.604)	1.600 (0.502)
CGI-G2	1.200 (0.615)	1.500 (0.606)
CGI-G3	1.400 (0.680)	1.450 (0.510)
CR-G1	0.400 (0.502)	0.850 (0.745)
CR-G2	0.550 (0.686)	1.250 (0.716)
CR-G3	0.350 (0.489)	0.950 (0.759)

Maximum mean micro-leakage in the crown-restoration interface was observed in CGI restorative material. Among the Glass ionomer, G1 showed the highest micro-leakage value at 1.450 (0.604), followed by G3 at 1.400 (0.680). The Nano-hybrid restorative composite showed the least mean micro-leakage at the crown- restoration interface. It was 0.400 (0.502), 0.550 (0.686) and 0.350 (0.489) for G1, G2 and G3, respectively. The highest mean micro-leakage at the Cementum-Restoration interface was observed for RMGI–G3 at 1.700 (0.571), followed by CGI–G1 with 1.6000 (0.502). CR–G1 recorded the lowest micro-leakage at this interface with a mean value of 0.850 (0.745).

The Kruskal–Wallis test (Table 3) was conducted to evaluate the difference in mean micro-leakage ranks between the restorations at both interfaces. The results showed the existence of a statistically significant difference in ranks among all three restorative materials at both interfaces. *P*-value for the Crown-restoration and Cementum-restoration interfaces were 0.0001 and 0.002, respectively. The lowest mean rank for micro-leakage at the crown-restoration interface was CR–G3 at 55.50 and CGI–G1 recorded the highest rank. The highest and lowest ranks at the Cementum-Tooth interface were recorded by RMGI- and CR-G1 at 115.30 and 58.73, respectively.

**Table 3:** Kruskal–Wallis analysis for mean microleakage of various groups.

Group	N	Mean rank	
		Crown-restoration	Cementum-restoration
RMGI-G1	20	91.90	96.70
RMGI-G2	20	88.93	95.15
RMGI-G3	20	87.88	115.30
CGI-G1	20	127.25	106.00
CGI-G2	20	111.85	99.80
CGI-G3 CR-G1	20 20	123.75 59.00	94.38 58.73
CR-G2	20	68.45	83.53
CR-G3	20	55.50	64.93
Total	180		

**Notes:**

*P* = 0.0001 for Crown-restoration; *P* = 0.002 at Cementum-Restoration interface.

S-significant 0.5% level of confidence.

The Pair-wise Man–Whitney (Tables 4 and 5) analysis indicated the statistically significant difference between CGI-G1, G2, and G3 with all CR groups with *P* ≤ 0.05 at Crown-Restoration interfaces. The CR-G1 had a significant difference with all other groups except RMGI-G3 (*p* = 0.073), CR-G2 (*p* = 0.576) and CR-G3 (*p* = 0.396) at the crown-restoration interface. Similarly, CR-G3 had a significant difference with all groups except between CR-G1and CR-G2.

**Table 4:** Pairwise comparisons using Mann–Whitney tests for each group.

Group	Crown-restoration interface							
	RMGI-G2	RMGI-G3	CGI-G1	CGI-G2	CGI-G3	CR-G1	CR-G2	CR-G3
RMGI-G1	0.835	0.794	0.016*	0.167	0.031*	0.023*	0.112	0.012*
RMGI-G2		0.930	0.007*	0.094	0.015*	0.030*	0.142	0.015*
RMGI-G3			0.017*	0.136	0.031*	0.073	0.233	0.043
CGI-G1				0.191	0.880	0.000*	0.000*	0.000*
CGI-G2					0.279	0.000*	0.004*	0.000*
CGI-G3						0.000*	0.001*	0.000*
CR-G1							0.576	0.747
CR-G2								0.396

**Note:**

* Significant at *P* ≤ 0.05.

**Table 5:** Pairwise comparisons using Mann–Whitney tests for each groups at Cementum-restoration interface.

Group	Cementum-restoration interface							
	RMGI-G2	RMGI-G3	CGI-G1	CGI-G2	CGI-G3	CR-G1	CR-G2	CR-G3
RMGI-G1	0.903	0.239	0.619	0.903	0.809	0.29	0.407	0.064
RMGI-G2		0.167	0.497	0.784	0.916	0.025*	0.451	0.062
RMGI-G3			0.389	0.219	0.085	0.000*	0.028*	0.001*
CGI-G1				0.661	0.348	0.002*	0.115	0.006*
CGI-G2					0.655	0.006*	0.261	0.020*
CGI-G3						0.009*	0.422	0.030*
CR-G1							0.092	0.673
CR-G2								0.205

**Note:**

* Significant at *P* ≤ 0.05.

At the Cementum-restoration interface, the result confirms the significant difference between CGI and CR material with the *p*-value *P* ≤ 0.05 except CR-G2. #RMGI-G2 and RMGI-G3 groups also had a significant difference between CR-G1 and CR-G3.

## Discussion

The long-term clinical performance of dental restorations is assessed with their ability for close adaptation to prepared cavities within tooth structure (Van Dijken, Pallesen & Benetti, 2019). A broader consensus exists among the researchers on an association of marginal gap and consequent micro-leakage with the initiation of secondary caries (Totiam et al., 2007; Borouziniat, Khaki & Majidinia, 2019). Hence, microleakage tests are employed to evaluate the effectiveness of restorations. Dental researchers suggest different methods to assess micro-leakage; it includes dye penetration, radioactive isotope infiltration, dye extraction and bacterial culture (AlHabdan, 2017). The micro-leakage evaluation with dye penetration has the advantage of being low cost, and direct observation could assess the depth of infiltration under the microscope (Heintze, 2007). The lower molecular weight and lesser diameter than most bacterial cells make the methylene blues the most preferred dye material. These molecular properties are helpful to determine the micro-leakage through the narrow marginal gap (De Almeida et al., 2003).

Secondary caries around indirect restorations is a commonly observed clinical situation. The structural defect at the cervical margin is a mixed lesion comprising the cementum, dentin, and indirect restoration margins (Nedeljkovic et al., 2020). Additionally, the squeezing effect experienced by cervical restorations (Lee & Eakle, 1996) during mastication makes the restorations at the crown margin unique from the restorations at other locations. This study result showed that all the tested restorative materials had a varying range of micro-leakage. The composite restorative materials had the least marginal leakage at both interfaces in all three cavity configurations, followed by RMGI and CGI.

Several researchers prefer the CGI restorations (Kampanas & Antoniadou, 2018; McDonough et al., 2002) at the cervical area due to their compatible modulus elasticity to overcome flexural stress and comparable coefficient of thermal expansion with tooth structure (Francisconi et al., 2009). An additional advantage of CGI is its sustained fluoride release and the semipermeable surface for salivary calcium and phosphate ions to facilitate the remineralization of enamel (Schwendicke et al., 2019). The absence of teeth conditioning by acid etching and primer application could be a reason for the lesser adaptation of CGI (Somani et al., 2016). Teeth conditioning with acid etching and primer helps in modifying the smear layer and facilitates the wetting of the tooth surface (Sofan et al., 2017). Hence, CGI bonding to tooth structure solely depended on ionic bonding, devoid of any micro-mechanical bonding. Abd El Halim & Zaki (2011) also reported the higher microleakage in CGI compared to light-cured, Nano-filled glass ionomer restorations. Pontes et al. (2014) described similar trends amongst CGI of having more micro-leakage than RMGI. The study results are also in corroboration with reports of the negative effect of long-term storage and thermocycling on the bonding strength and physical properties of CGI (Ratih, Palamara & Messer, 2006). The micro-leakage at the crown-restoration margin was also higher in the CGI compared to other restorations. Reis et al. (2004) observed the ineffectiveness of air-abrasion on the micro-leakage of CGI restoration. It could be due to a lack of chemical bonding to the metal substrate and incomplete wetting of the metal surface because of its higher viscosity.

The RMGI was developed to overcome the limitations in mechanical properties, setting time, and moisture contamination of CGI. They comprise light-activated metacrilate and resin HEMA or Bis-GMA besides CGI. In the present study, 37% phosphoric acid etching conditioned the tooth structure, and Scotch bond Universal Adhesive was applied before RMGI restoration. The study results showed the significantly lesser micro-leakage compared to CGI at both crown- restoration and tooth-restoration interfaces. The results agreed with previous studies recommending tooth conditioning with primers to improve the marginal integrity at cervical cavities (Shafiei & Akbarian, 2014; Rocha et al., 2018). Phosphoric acid etching aids in increasing surface area for bonding and enhances the wettability of the tooth surface. Scotch bond Universal comprises methacryloxydecyl dihydrogen phosphate (MDP), methacrylate resins; they chemically bond to hydroxyapatite crystals (Yoshida et al., 2004). Micro-mechanical bonding and polyalkenoate-hydroxyapatite chemical bonding assist the RMGI bonding to the tooth substrate (Sidhu & Nicholson, 2016). Collective bonding for both micro-mechanical and chemical bonding could be attributed to improved adaptation and lesser hydrolytic degradation during thermocycling (Stockton & Tsang, 2007). Nevertheless, earlier researchers report polymerization shrinkage up to 3% (Berzins et al., 2010) and water uptake (Beriat & Nalbant, 2009) by RMGI material. These attributes of RMGI compromise its bonding durability with tooth structure. However, De Magalhaes, Serra & Rodrigues Junior (1999) reported no significant difference in micro-leakage values of CGI, RMGI, and CR. The contradictory results could be due to the difference in methodology and assessment methods.

The composite resin restorations incorporate Bis-GMA or urethane dimethacrylate resin matrix and an inorganic filler. The MDP primer application subsequent to phosphoric acid etching facilitates better bonding to the tooth structure (Alqahtani, 2015). Both RMGI and Composite resin restorations exhibited lower microleakage values at crown-restoration margins. The silane component in primer improved the adhesion to the metal substrate, and the air-borne roughening with aluminum oxide aid in the micro-mechanical bonding (Matinlinna, Lung & Tsoi, 2018). The Scotch bond Universal comprises MDP, methacrylate resins, ethanol, water, and a functional copolymer of polyacrylic and polyitaconic acids. The MDP allows for better adhesion performance to enamel, improved product stability, adhesion to metal, and non-glass ceramic substrates (Carrilho et al., 2019). Presences of ethanol within the primer enhance the wetting of the dentin and metal surface (Alex, 2015). As a result, it will assist in the infiltration of adhesive into the demineralized dentin and to maintain the expanded collagen network (De Souza, Di Nicoló & Bresciani, 2018).

The micro-leakage values at the Cementum-restoration interface were higher in all the tested groups. The result agreed with reports from earlier researches of increased micro-leakage at the cementum margin compared to enamel surfaces (Ludlow et al., 2014; Beznos, 2001). The structural differences of cementum from enamel and dentin include the acellular extrinsic fiber and the absence of patent tubule orifices. Hence, affecting its bonding capability. The phosphoric acid conditioning may lead to diminished hydroxyapatite from the etched dentin surface at the root surface area. Consequently, it could cause the depletion of the ionic bond between the carboxyl group of RMGI and composite with hydroxyapatite (Borouziniat, Khaki & Majidinia, 2019). The authors suggest the utilization of a Nano-hybrid composite along with metal and teeth conditionings to restore secondary caries at indirect restoration margins. The composite restoration could be employed as an interim measure until the replacement of the crown to prevent further damage to the tooth structure.

Caries induction methods by demineralizing chemical are criticized as the process lack saliva and biofilm. The chemical caries induction technique was reported to produce superficial lesions. However, the researchers showed the histological resemblance to natural root caries with an outer well-mineralized layer succeeded by an area of increased demineralization (McIntyre, Featherstone & Fu, 2000).

Moreover, the chemically induced demineralized lesions were reported with similar hardness values with natural caries lesions. Hence researchers recommend the chemical methods to achieve the dentin substrate layer simulating structure post caries removal (Marquezan et al., 2009).

This study tested the material in vitro. Hence, extrapolating to the clinical situation requires clinical testing. The microleakage evaluation by visual scoring is a qualitative assessment involving considerable subjectivity and questionable reproducibility. Thus, several researchers utilize alternative methods like a digital appraisal with image tool software, radioactive isotopes, Microcomputed tomography, confocal laser scanning microscopy, and Optical coherence tomography. The study did not examine the effect of occlusal loading on the micro-leakage values. The noxious stimuli secondary caries led to reparative changes in dentin; these changes could also lead to different outcomes. The authors suggest clinical studies to assess the long-term deterioration of these bonding interfaces in a highly complex and dynamic environment of the oral cavity.

## Conclusion

All the restorations tested in the study showed a varying amount of interfacial micro-leakage, none of the tested restorations could completely prevent the micro-leakage. The hybrid composite restoration with universal adhesive and air abrasion of the exposed metal margin showed the least microleakage value. The conventional glass ionomer restorations showed the highest micro-leakage at the crown-restoration margin, while RMGI recorded higher marginal leakage at the cementum-restoration margin. The clinical implication of the study is the potential utilization of hybrid composite to restore the marginal gap at cervical margins of full coverage restorations. The micro-leakage values at the cementum-restoration margins were more than the crown-restoration margin in all the evaluated restorations.

## Supplemental Information

10.7717/peerj.10823/supp-1 Supplemental Information 1 Dataset.

## Additional Information and Declarations

### Data Availability

The following information was supplied regarding data availability: Raw measurements are available as a Supplemental File.
